# Species-Specific Differences in the Susceptibility of Fungi to the Antifungal Protein AFP Depend on C-3 Saturation of Glycosylceramides

**DOI:** 10.1128/mSphere.00741-19

**Published:** 2019-12-11

**Authors:** Norman Paege, Dirk Warnecke, Simone Zäuner, Silke Hagen, Ana Rodrigues, Birgit Baumann, Melanie Thiess, Sascha Jung, Vera Meyer

**Affiliations:** a Technische Universität Berlin, Institute of Biotechnology, Chair of Applied and Molecular Microbiology, Berlin, Germany; b Institute of Plant Science and Microbiology, University of Hamburg, Hamburg, Germany; Carnegie Mellon University

**Keywords:** antimicrobial peptide, antifungal protein AFP, gamma core, damage-response framework, sphingolipid, glycosylceramide, Δ3(E)-desaturase, filamentous fungus, yeast

## Abstract

AFP is an antimicrobial peptide (AMP) produced by the filamentous fungus Aspergillus giganteus and is a very potent inhibitor of fungal growth that does not affect the viability of bacteria, plant, or mammalian cells. It targets chitin synthesis and causes plasma membrane permeabilization in many human- and plant-pathogenic fungi, but its exact mode of action is not known. After adoption of the “damage-response framework of microbial pathogenesis” regarding the analysis of interactions between AMPs and microorganisms, we have recently proposed that the cytotoxic capacity of a given AMP depends not only on the presence/absence of its target(s) in the host and the AMP concentration applied but also on other variables, such as microbial survival strategies. We show here using the examples of three filamentous fungi (Aspergillus niger, Aspergillus fumigatus, and Fusarium graminearum) and two yeasts (Saccharomyces cerevisiae and Pichia pastoris) that the important parameters defining the AFP susceptibilities of these fungi are (i) the presence/absence of glycosylceramides, (ii) the presence/absence of Δ3(*E*) desaturation of the fatty acid chain therein, and (iii) the (dis)ability of these fungi to respond to AFP inhibitory effects with the fortification of their cell walls via increased chitin and β-(1,3)-glucan synthesis. These observations support the idea of the adoption of the damage-response framework to holistically understand the outcome of AFP inhibitory effects.

**IMPORTANCE** Our data suggest a fundamental role of glycosylceramides in the susceptibility of fungi to AFP. We discovered that only a minor structural difference in these molecules—namely, the saturation level of their fatty acid chain, controlled by a 2-hydroxy fatty N-acyl-Δ3(*E*)-desaturase—represents a key to understanding the inhibitory activity of AFP. As glycosylceramides are important components of fungal plasma membranes, we propose a model which links AFP-mediated inhibition of chitin synthesis in fungi with its potential to disturb plasma membrane integrity.

## Abstract (precis)

Our data suggest a fundamental role of glycosylceramides in the susceptibility of fungi to AFP. We discovered that only a minor structural difference in these molecules—namely, the saturation level of their fatty acid chain, controlled by a 2-hydroxy fatty N-acyl-Δ3(*E*)-desaturase—represents a key to understanding the inhibitory activity of AFP. As glycosylceramides are important components of fungal plasma membranes, we propose a model which links AFP-mediated inhibition of chitin synthesis in fungi with its potential to disturb plasma membrane integrity.

## INTRODUCTION

The continuous rise of plant-, animal-, and human-pathogenic fungi that are virtually resistant to the antifungal drugs currently used calls urgently for novel antifungal substances and new antifungal strategies (1). In addition, most of the antifungal drugs used exert cytotoxic effects also on humans because their target molecules are present not only in fungal cells but also in higher eukaryotes, including humans (2). The discovery of novel antifungals with specific targets that are present only in fungi is, thus, one important prerequisite for the development of new fungus-specific drugs. An interesting lead compound for a new generation of fungicides is the antifungal protein AFP from Aspergillus giganteus. This is not only because of its fungus-specific mode of action in the micromolar range but also because of its high stability under conditions of temperature (80°C), pH (ranging from pH 2 to pH 10), and protease stresses (3). It has furthermore been demonstrated that treatment of plants used agriculturally with AFP or transgenic expression of the *afp* gene in these plants can protect them against fungal infections caused by Blumeria graminis, Fusarium oxysporum, and Magnaporthe grisea (Pyricularia grisea), to name but a few (3–5).

AFP is a small (51 amino acids) cationic and amphipathic peptide whose three-dimensional structure has been resolved by nuclear magnetic resonance (NMR) (4). It is the founder molecule of the AFP family, which consists of more than 50 members present in about 30 different *Ascomycota* (6). The structural characteristics of this family include a γ-core motif, six conserved cysteine residues, and a highly stable beta-barrel folding. Notably, the γ-core is a common feature in all cysteine-stabilized antimicrobial peptides (AMPs) from bacteria, fungi, plants, and (in)vertebrates (7). It has recently been demonstrated by *in silico* molecular dynamics simulations that AFP interacts strongly with a fungal membrane model without penetrating it, whereby its γ-core motif is actively involved in the formation of the membrane-AFP binding interface (4). This agrees with electron microscopic data showing that AFP binds heavily to the cell wall and plasma membrane of sensitive fungi (e.g., A. niger) but not to the cell surface of AFP-resistant fungi (e.g., Penicillium chrysogenum). In fact, AFP binds to chitin *in vitro* and has been shown to inhibit chitin biosynthesis *in vivo* (8). Chitin is directly adjacent to the plasma membrane and an important structural component of the fungal cell wall together with β-(1,3)-glucan, β-(1,6)-glucan, α-(1,3)-glucan, (galacto)mannans, and glycoproteins (9). In addition to its ability to disturb chitin biosynthesis in susceptible fungi, AFP has also been shown to stretch and permeabilize their plasma membranes within minutes after application (8, 10, 11).

Fungal chitin biosynthesis is far from being understood. It is assumed that chitin is synthesized at the plasma membrane by the transmembrane-localized chitin synthases (CHSs) that are transported to the plasma membrane in an inactive form within chitosomes (a specific population of secretory vesicles) and that become activated after plasma membrane insertion (9). Yeast and filamentous fungal genomes contain several CHS-encoding genes (up to 12 per genome) that are thought to fulfill different functions during growth and developmental processes (12). The three-dimensional structure has been resolved for none of the CHSs so far; hence, it is currently impossible to predict or deduce any direct interaction of AFP with a CHS based on structural information. Thus, a number of possible scenarios have been proposed which could explain the inhibitory effect of AFP being localized at the outer side of the fungal plasma membrane and exerting effects on both chitin biosynthesis and plasma membrane integrity (3) as follows: (i) AFP might prevent the fusion of chitosomes with the plasma membrane; (ii) AFP might disturb the proper embedding of CHSs in the plasma membrane, for example, by interacting with adjacent plasma membrane components; and/or (iii) AFP might interfere with the enzymatic activity of CHSs, for example, by binding to newly synthesized chitin, thus preventing polymerization of the nascent chain. All three events are conceivable and are not mutually exclusive. They would eventually cause membrane stretching due to malformation of the cell wall, which would no longer be able to withstand the internal turgor pressure. This, in turn, would lead to cell lysis, predominantly at the hyphal tips where cell wall biosynthesis mainly occurs. In agreement, tip-localized bursting is frequently observed in the highly AFP-susceptible species A. niger when treated with AFP (8, 10, 11).

Fungi use different survival strategies to fight against any lethal effects of AFP. Analysis of different wild-type strains and cell wall mutants of Saccharomyces cerevisiae, A. niger, and Fusarium oxysporum uncovered the fact that fungi which are less susceptible to AFP fortify their cell walls with chitin in response to AFP, whereas fungi which are more susceptible to AFP fail to do so (13). This was mechanistically explained by the observation that the less susceptible strains rely on the calcium/calcineurin/Crz1p signaling pathway to reinforce chitin synthesis, whereas the susceptible strains deploy the Pkcp/Slt2p/Rlm1p cell wall integrity pathway, whose main output is glucan and not chitin synthesis (13). This observation led us to propose the adoption of the “damage-response framework of microbial pathogenesis” (14–16) to explain the effect of AFP on fungi. In translating the tenets of this conceptual approach to AFP-fungus interactions, it can be postulated that the outcome of an AFP attack is dependent on (i) the innate susceptibility of the microorganism, (ii) the damage potential of AFP defined by its concentration and (non-)target-specific molecular interactions, and (iii) the survival response, which can be appropriate or too weak or too strong and thus detrimental to the host. Consequently, the adopted damage-response framework predicts that the cytotoxic capacity of AFP on fungi is contingent on various factors (13).

**FIG 1:**
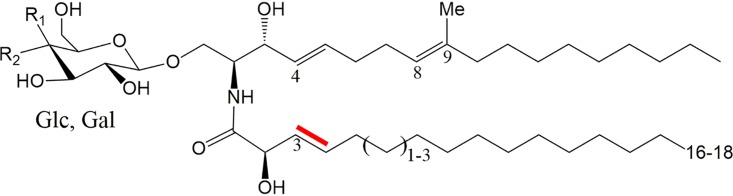
Fungal glycosylceramides consist of either a glucose (Glc) or a galactose (Gal) moiety, a sphingoid base, and a fatty acid. The double bond generated by the 2-hydroxy fatty N-acyl-Δ3(*E*)-desaturase is marked in red.

In order to further study the fungus-specific mode of action of AFP, the different susceptibilities of fungi to AFP, and the link between its cell wall effects and its cell membrane effects, we searched the literature for plasma membrane components which are not present in prokaryotes but which are present in eukaryotes and which, most importantly, differ between eukaryotic kingdoms. This is the case for sphingolipids, which are present in eukaryotes but differ with respect to their composition in different kingdoms (17). Glycosylceramides (GlyCer) are very simple forms of sphingolipids which are prevalent in fungi (Fig. 1). They consist of a sphingoid base, a fatty acid, and either a glucose (GlcCer) or galactose (GalCer) moiety. Fungal GlyCer can be distinguished from those in plants and mammals by a methyl group branching from C9 of the sphingoid base. In addition, there are variable levels of unsaturation and lengths of the fatty acid chain (Fig. 1) (18). A further literature survey for data on fungi whose GlyCer composition has been resolved and for which the MIC of AFP is known uncovered that, surprisingly, the susceptibility of fungi to AFP seemed to match the presence or absence of an Δ3(*E*) double bond within the fatty acid (Fig. 1 and Table 1). The highly susceptible species A. niger, for example, contains unsaturated GlyCer, the less susceptible species A. nidulans contains saturated GlyCer, and the AFP-resistant species S. cerevisiae does not contain GlyCer at all. Importantly, the enzyme synthesizing the unsaturation at the C-3 atom [2-hydroxy fatty N-acyl-Δ3(*E*)-desaturase] has been studied in Fusarium graminearum (19). In the present study, we therefore experimentally investigated differences in the levels of AFP susceptibility of different fungi (A. niger, A. fumigatus, F. graminearum, Pichia pastoris [new name, Komagataella phaffii], and S. cerevisiae) dependent on the presence or absence of GlyCer and their saturation levels. In doing so, we followed knockout and knock-in approaches with different orthologous genes predicted to encode a Δ3(*E*)-desaturase and additionally performed different chemical assays to inhibit GlyCer biosynthesis in these fungi. The data presented here demonstrate a direct correlation between the (un)saturation state of GlyCers and fungal susceptibilities to AFP.

**TABLE 1:** Literature data for fungal glycosylceramides and MICs of AFP^*a*^

Fungus	GlyCer	Δ3 unsaturation	MIC (μg/ml)
Aspergillus niger	GlcCer	Present	1
	GalCer	Present	

Aspergillus fumigatus	GlcCer	Present	10
	GalCer	Present	

Fusarium graminearum	GlcCer	Present	10

Fusarium solani	GlcCer	Present	120

Aspergillus nidulans	GlcCer	Absent	200

Saccharomyces cerevisiae		Absent	NE

Candida albicans	GlcCer	Absent	NE

a Fungal glycosylceramide data are from reference 18; AFP MIC data are from reference 11. GlcCer, glucosylceramide; GalCer, galactosylceramide; NE, no effect.

## RESULTS

### Fungal glucosylceramides mediate sensitivity to AFP.

**FIG 2:**
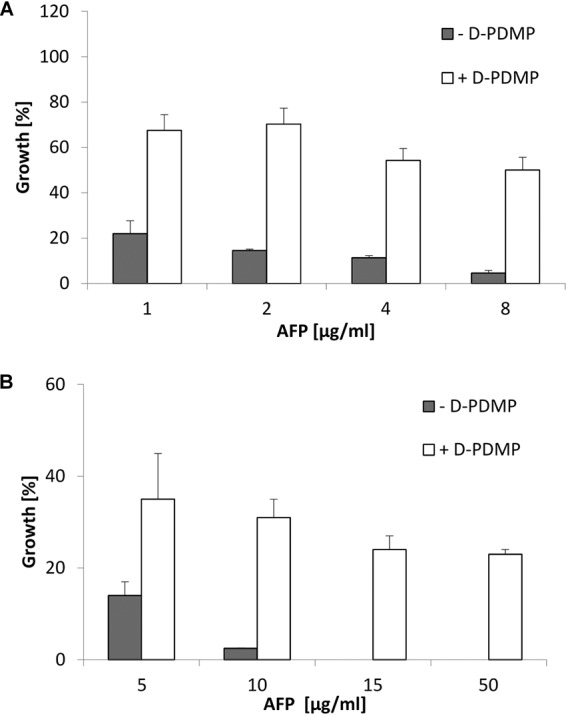
The AFP susceptibility of A. niger and A. fumigatus is dependent on glucosylceramides. (A and B) Growth of (A) A. niger wild-type strain N402 and (B) A. fumigatus wild-type strain ATCC 9197 at different AFP concentrations in the presence (white bars) or absence (gray bars) of the GlcCer synthesis inhibitor d-PDMP. Growth was evaluated by determining the optical density at 600 nm in microtiter plate cultivations. Data were normalized to control cultures which were not treated with AFP. Data are mean values referring to two biological replicates, each measured as a technical triplicate. Error bars express standard deviations.

The glucosylceramide biosynthetic pathway has been shown to be important for spore germination and hyphal growth in A. nidulans, A. fumigatus, and F. graminearum (20–22). A central position in this pathway is adopted by the glucosylceramide synthase GCS, which transfers a glucose moiety from uridine 5-diphosphate (UDP)-glucose onto the C1 hydroxyl group of the ceramide. In a preliminary experiment, we chemically inhibited GCS activity in AFP-susceptible strains of A. niger and A. fumigatus using d-threo-1-phenyl-2-decanoylamino-3-morpholino-1-propanol (d-PDMP) to determine whether GlcCers might play a role in the susceptibility of filamentous fungi to AFP. d-PDMP is a synthetic analogue of ceramide which acts as an antimetabolite and inhibits the covalent bonding of ceramides with glucose. This inhibition, in turn, leads to the absence of GlcCers in filamentous fungi, as shown previously for A. nidulans (23). As depicted in Fig. 2, d-PDMP treatment considerably decreased the susceptibility of A. niger and A. fumigatus to AFP, as the survival rates increased substantially (>3-fold). These data suggest that the presence of GlcCers might indeed play a role in defining the susceptibility of both strains to AFP. However, the addition of d-PDMP did not completely rescue the strains and its protective effect was stronger for A. niger than for A. fumigatus, potentially pointing at strain-specific differences in innate susceptibilities and/or survival responses. Note that the protective effect of d-PDMP decreased with the increasing AFP concentration, implying that GlcCers might not be the only targets of AFP.

### Δ3(*E*)-desaturase gene expression mediates sensitivity to AFP.

Encouraged by this preliminary experiment, we tested if the Δ3(*E*) desaturation of GlyCer can affect AFP susceptibilities in different fungi. As summarized in Table 1, we hypothesized that a causal relationship between the Δ3(*E*) unsaturation of GlyCer and the susceptibility of fungi to AFP might exist. In order to study this in detail, we wanted to identify the gene encoding the Δ3(*E*)-desaturase in AFP-susceptible A. niger to generate a knockout strain for comparative susceptibility assays. Complementarily to this approach, we wanted to establish knock-in strains in otherwise AFP-resilient strains to test whether the introduction of a gene encoding the Δ3(*E*)-desaturase can switch the resistance state to susceptibility.

BLAST analyses using the known Δ3(*E*)-desaturase gene from F. graminearum (open reading frame [ORF] code FJ176922.1) identified only one orthologous gene in A. niger, with sequence identity of 61% at the amino acid level (ORF code An01g09800). A search of the PFAM database for conserved domains revealed a relationship with the fatty acid desaturase superfamily (accession no. pfam00478; E value 6.8e−11). For brevity, we named this gene *dtdA* (delta three desaturase). Analysis of our in-house A. niger transcriptomic database, containing expression data for ∼14,000 A. niger genes subjected to 155 different cultivation conditions, revealed that *dtdA* was expressed under nearly all cultivation conditions, albeit at very low levels similar to those seen with regulatory Rho-GTPases (24) (see Fig. S1 in the supplemental material).

10.1128/mSphere.00741-19.1 FIG S1 Δ3(*E*)-desaturase expression levels under 155 different A. niger cultivation conditions. Mean transcript levels for An01g09800 are shown based on genome-wide microarray data published by Schäpe et al. (P. Schäpe, M. J. Kwon, B. Baumann, B. Gutschmann, et al., 2019, Nucleic Acids Res. 47:559–569). Download FIG S1, TIF file, 1.0 MB. Copyright © 2019 Paege et al. 2019 Paege et al. This content is distributed under the terms of the Creative Commons Attribution 4.0 International license.

**FIG 3:**
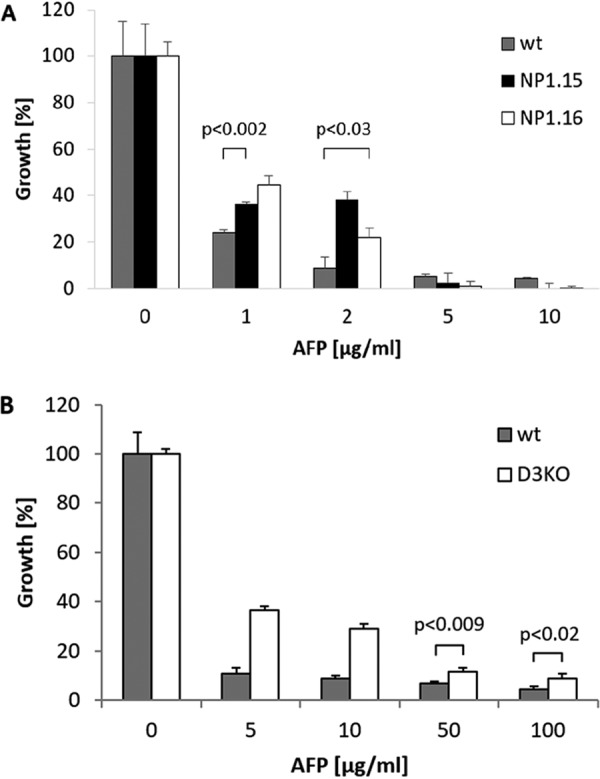
A. niger and F. graminearum deleted for the Δ3(*E*)-desaturase-encoding gene show reduced AFP susceptibilities. (A) Growth of A. niger wild-type (wt) strain N402 (gray bars) and Δ*dtdA* strains NP1.15 (black bars) and NP1.16 (white bars) at different AFP concentrations. (B) Growth of F. graminearum wild-type (wt) strain (gray bars) and Δ3(*E*)-desaturase knockout strain (D3KO; white bars). Growth was evaluated by determining the optical density at 600 nm in microtiter plate cultivations. Data were normalized to the growth, i.e., 100% growth, of control samples not treated with AFP. Data are from two independent experiments, each performed as a technical triplicate. Error bars express standard deviations. *P* values indicate significance of those values where the differences, with respect to the corresponding error bars, were low.

Thus, we generated *dtdA* deletion strains in A. niger (strains NP1.15 and NP1.16) and compared their susceptibilities to that of the wild-type strain (strain N402). In addition, we performed an analogous comparison with a wild-type strain of F. graminearum (strain 8/1) and its Δ3(*E*)-desaturase deletion derivative (strain Δ3FgKO), which had been established previously (19). As depicted in Fig. 3, all deletion strains displayed substantially improved growth in the presence of AFP compared to their parental strains, demonstrating that the strains are less susceptible to AFP when their Δ3(*E*)-desaturase genes are inactive. As A. niger strains NP1.15 and NP1.16 were basically indistinguishable with respect to growth and AFP susceptibility (Fig. 3), further analyses were conducted with strain NP1.15 only.

We next transformed the AFP-resistant yeast P. pastoris with Δ3(*E*)-desaturase genes from A. niger and F. graminearum, giving strain BBA21.3 (expressing the A. niger Δ3(*E*)-desaturase gene) and strain SZ51 (expressing the F. graminearum Δ3(*E*)-desaturase gene), respectively. Both strains became susceptible to high AFP concentrations when the *dtdA* gene was expressed, which was not the case in the corresponding controls. The growth rate of strain BBA21.3 (SZ51) was reduced by 40% (25%) in the presence of 100 μg/ml AFP when it expressed the Δ3(*E*)-desaturase gene. The presence (absence) of Δ3(*E*)-desaturase gene transcripts under inducing (noninducing) cultivation conditions was proven by reverse transcription-quantitative PCR (qRT-PCR) (Fig. S2 and data not shown).

10.1128/mSphere.00741-19.2 FIG S2 A. niger Δ3(*E*)-desaturase transcript expression levels in wild-type and Δ3(*E*)-desaturase mutant strains of filamentous fungi and yeast. Threshold cycle (*C_T_*) values are shown as a measure of the transcript expression levels of Δ3(*E*)-desaturase normalized to that of the actin housekeeping gene analyzed by qRT-PCR. The presence of transcript levels is shown by *C_T_* ratios in the range of around 1 to 1.2 and their absence by *C_T_* ratios in the range of 2 to 2.6. Data were derived from two independent experiments. (A) Samples derived from cultures incubated at 30°C. (B) Samples derived from A. niger wild-type strain N402 cultivated at different temperatures to analyze the temperature dependence of Δ3(*E*)-desaturase expression. Desaturase (+), gene expressed; Desaturase (−), gene not expressed. Download FIG S2, TIF file, 0.5 MB. Copyright © 2019 Paege et al. 2019 Paege et al. This content is distributed under the terms of the Creative Commons Attribution 4.0 International license.

A further experiment verified that both the presence of GlyCer and expression of the Δ3(*E*)-desaturase gene are fundamental prerequisites for fungal AFP susceptibility. As S. cerevisiae lacks GlyCer (19), one would expect that expression of a Δ3(*E*)-desaturase gene in this AFP-resistant strain would not make it AFP susceptible, because the substrate for the enzyme is not synthetized by S. cerevisiae. Indeed, when the *dtdA* gene of A. niger was constitutively expressed in S. cerevisiae (BBA20.2), the strain did not show any reduction of growth in the presence of 100 μg/ml AFP, despite high mRNA transcript levels of the *dtdA* gene (Fig. S3 and data not shown).

10.1128/mSphere.00741-19.3 FIG S3 Susceptibility of S. cerevisiae to AFP in presence and absence of Δ3(*E*)-desaturase expression. The growth characteristics of S. cerevisiae strains BY4741 (wild type) and BBA20.2 [Δ3(*E*)-desaturase knock-in] were tested, according to a previously published method (13), in the presence of 400 μg/ml AFP or without the peptide. Cultivations were carried out in technical duplicate in microtiter plate format and repeated three times. Error bars express standard deviations. Δ3(*E*)-desaturase (+), gene expressed; Δ3(*E*)-desaturase (−), gene not expressed. Data are expressed as means of results from two independent experiments each performed in triplicate. Error bars express standard deviations. Δ3(*E*)-desaturase (+), gene expressed; Δ3(*E*)-desaturase (−), gene not expressed. Download FIG S3, TIF file, 0.3 MB. Copyright © 2019 Paege et al. 2019 Paege et al. This content is distributed under the terms of the Creative Commons Attribution 4.0 International license.

### DtdA mediates desaturation of GlyCer fatty acid chains.

**FIG 4:**
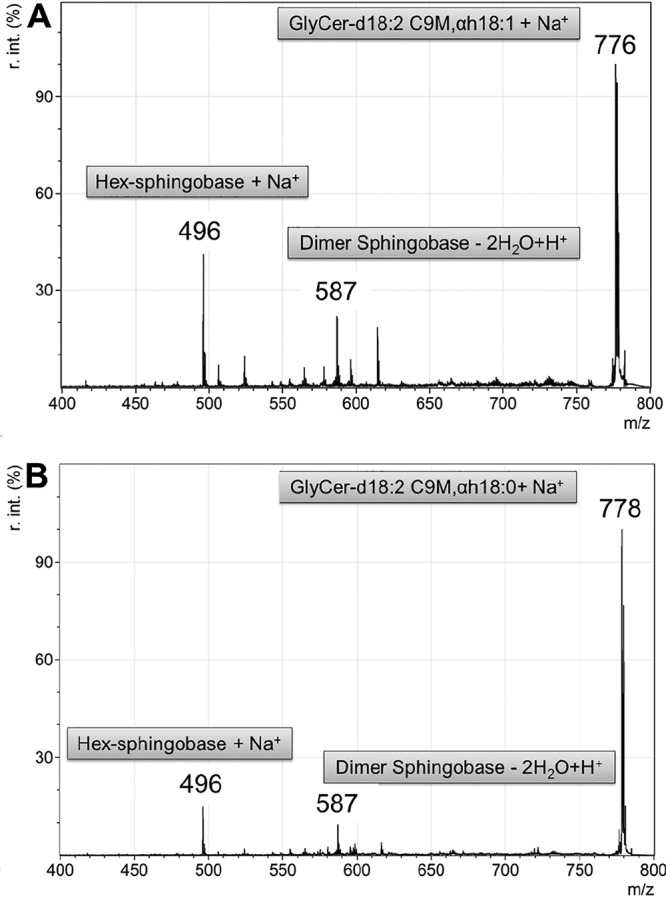
MS/MS analysis of A. niger wild-type and its *dtdA* deletion strain. Both panels show an overlay of MS spectra for detection of the parental GlyCer ions and the fragmented parental ion masses. (A) Wild-type strain N402. R. int. (%), relative percent intensity. (B) *dtdA* deletion strain NP1.15. Hex, hexose.

Mass spectrometry (MS) analyses proved that the *dtdA* gene of A. niger encodes a protein exerting an Δ3(*E*)-desaturase enzymatic activity. The GlyCers were isolated and purified by thin-layer chromatography from the A. niger wild-type and its *dtdA* deletion strain and from the P. pastoris wild-type and its Δ3(*E*)-desaturase knock-in derivatives, the latter cultivated under inducing and noninducing conditions (Fig. 4 and Table 2). The unsaturation of the fatty acyl moiety of GlyCer was detected indirectly by measurement of the total mass of the purified GlyCers. The mass of the molecular ion [M + Na^+^] (776 *m/z*) corresponded to the calculated mass of 753 Da for C_43_H_79_O_9_N plus 23 Da for Na^+^. This is in agreement with the presence of Δ3(*E*)-unsaturated GlyCer, *N*-(*R*)-2´-hydroxy-(3*E*)-octadec-3-enoyl-1-*O*-β-d-hexosyl-(4*E*,8*E*)-9-methyl-sphinga-4,8-dienine (C43H79O9N), whereas the [M + Na^+^] of the Δ3-saturated counterpart was 778 *m/z* (corresponding to 755 Da for C_43_H_81_O_9_N plus 23 Da for Na^+^). Subsequent tandem MS (MS/MS) analysis led to the fragmentation of the parental ions into the hexosyl-sphingobase fragment [M + Na^+^] 496 *m/z* and a dimer of the sphingobase fragment [M -2 H_2_O + H^+^] 587 *m/z*. Neither fragment was affected by Δ3(*E*) unsaturation; therefore, both showed a constant molecular mass, regardless of their parental ions of [M + Na^+^] 776 *m/z* and 778 *m/z*, respectively. Thus, the mass differences of the parental ions can be explained only by mass differences of the fatty acid moieties due to their unsaturation state. As summarized in Table 2 (see also Fig. S4), all fungal strains lacking or not expressing a Δ3(*E*)-desaturase gene showed the [M + Na^+^] 778 *m/z* signal, which reflects the absence of the double bond in the fatty acid moiety in GlyCer. In contrast, strains expressing a Δ3(*E*)-desaturase gene displayed the [M + Na^+^] 776 *m/z* signal.

**TABLE 2:** MS/MS analysis of fungal strains expressing or lacking a Δ3(*E*)-desaturase gene^*a*^

Strain	Δ3(*E*)-desaturase	*m*/*z* GlyCer [M + Na^+^]	*m*/*z* hexose-sphingobase fragment [M + Na^+^]
P. pastoris BBA21.3 (induced)	Present	776 (∼15%), 778 (∼85%)	496, 496
P. pastoris BBA21.3 (not induced)	Absent	778	496
P. pastoris NP7.1 (not induced)	Absent	778	496
P. pastoris NP7.1 (induced)	Absent	778	496
A. niger NP1.15 (*ΔdtdA*)	Absent	778	496
A. niger N402 (18°C)	Present	776	496
A. niger N402 (30°C)	Present	776	496
A. niger N402 (37°C)	Present	776 (∼75%), 778 (∼25%)	496, 496

a *m*/*z*, mass-to-charge ratio.

10.1128/mSphere.00741-19.4 FIG S4 MS/MS analysis of glycosylceramides in A. niger and P. pastoris. MS spectra were determined for the detection of the parental GlyCer ions for A. niger N402 at 18°C (C; the spectrum is comparable to that seen at 30°C [not shown]) and 37°C (E) and for P. pastoris BBA21.3 (not induced [G] and induced [I]). Data representing overlay of the parental ion masses and their fragments are shown as follows: N402 at 18°C (D) and 37°C (F); BBA21.3 not induced (H) and induced (J). R. int. (%) = relative percent intensity; Hex, hexose. Download FIG S4, TIF file, 2.1 MB. Copyright © 2019 Paege et al. 2019 Paege et al. This content is distributed under the terms of the Creative Commons Attribution 4.0 International license.

Although the MS method used is not suitable for absolute quantification of the compounds, it is possible to determine the relative proportions of structurally very similar compounds by comparing their peak intensities. Thus, expression of the Δ3(*E*)-desaturase genes in P. pastoris did not reach 100% unsaturation levels. The desaturation efficiency of the A. niger *dtdA* gene reached an unsaturation level of about 15% (Fig. S4), whereas the desaturation efficiency of its ortholog from F. graminearum was 50% to 100% (19).

Because the enzyme activity of the F. graminearum Δ3(*E*)-desaturase has been described previously as temperature dependent, with only 20% enzyme activity at 30°C but 100% at 18°C (19), we tested whether this is also the case for DtdA. A. niger wild-type strain N402 was thus cultivated at 18, 30, and 37°C, and the *dtdA* transcript levels were analyzed by qRT-PCR (Fig. S2) and MS/MS data recorded for GlyCer populations purified from these biomass samples (Fig. S4). As summarized in Table 2, DtdA displayed 100% activity at 18 and 30°C and about 75% at 37°C, demonstrating that its temperature-dependent activity is less pronounced than and even reciprocal with respect to that of its F. graminearum ortholog.

### DtdA prevents strong cell wall reinforcement in A. niger and P. pastoris in response to AFP.

**FIG 5:**
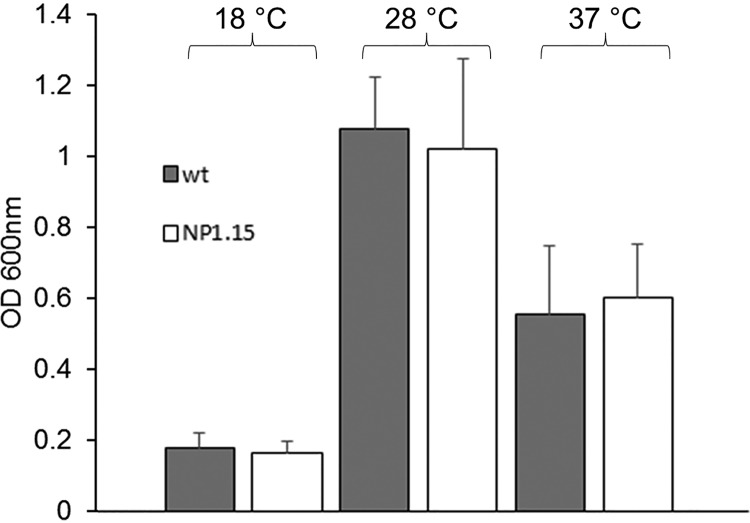
Biomass accumulation of A. niger wild-type N402 and its *dtdA* deletion strain. Cultures of A. niger wild-type (wt) strain N402 and Δ*dtdA* strain NP1.15 (white bars) were incubated, according to a method described in reference 8, in microtiter plates at three different temperatures for 48 h. Growth was evaluated by determining the optical density (OD) at 600 nm. Data were derived from three independent experiments each performed as a technical triplicate. Error bars express standard deviations.

Deletion of *dtdA* did not have any obvious consequences for hyphal growth, biomass accumulation, or sporulation of A. niger under conditions of cultivation in liquid or on solid medium (Fig. 5 and data not shown). Similarly, the presence or absence of the *dtdA* gene did not affect the levels of chitin and β-1,3-glucan in the cell wall of A. niger (Fig. S5), suggesting that the cellular role of DtdA for growth is rather negligible.

10.1128/mSphere.00741-19.5 FIG S5 Relative chitin and β-1,3-glucan content levels in A. niger wild-type strain (N402) and *ΔdtdA* deletion strain (NP1.15). The relative percentages of chitin and β-1,3-glucan in relation to the control strains, whose levels were set to 100%, are shown. Download FIG S5, TIF file, 0.3 MB. Copyright © 2019 Paege et al. 2019 Paege et al. This content is distributed under the terms of the Creative Commons Attribution 4.0 International license.

**FIG 6:**
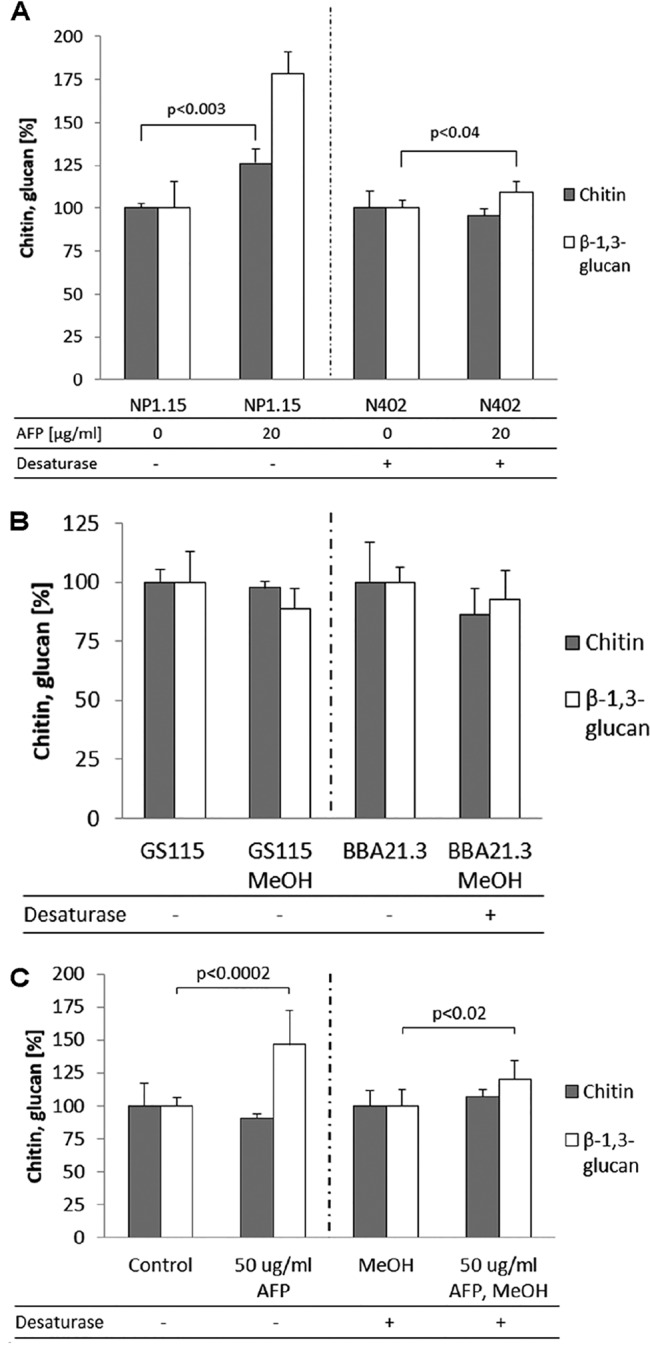
Chitin and β-1,3-glucan responses of A. niger and P. pastoris under conditions of AFP stress. The relative percentages of chitin and β-1,3-glucan in relation to the control strains, whose levels were set to 100%, are shown. (A) A. niger wild-type strain N402 and *dtdA* deletion strain NP1.15 in the presence or absence of 20 μg/ml AFP. (B) P. pastoris wild-type GS115 and *dtdA* knock-in strain BBA21.3 in the presence or absence of methanol as an inductor of *dtdA* expression. This experiment served as a control to prove that the two *Pichia* strains showed comparable levels of cell wall compound synthesis in the presence of methanol. (C) P. pastoris *dtdA* knock-in strain BBA21.3 in the presence and absence of AFP and methanol as an inductor of *dtdA* expression. Data are expressed as means of results from two independent biological replicates each performed as a technical triplicate. Error bars express standard deviations. *P* values indicate significance of values where the differences, with respect to the corresponding error bars, were low. Desaturase (+), *dtdA* expressed; Desaturase (−), *dtdA* not expressed.

However, the cell wall stress responses of A. niger to the presence of AFP differed significantly between the wild-type strain N402 and its *dtdA* deletion derivative, strain NP1.15. Whereas the AFP-susceptible N402 strain responded to AFP-provoked cell wall stress with an increase in β-1,3-glucan (+10%; the chitin level remained constant), the less AFP-susceptible Δ*dtdA* NP1.15 strain was able to reinforce its cell wall with both chitin (+26%) and β-1,3-glucan (+78%) upon AFP treatment (Fig. 6A). Hence, there is a positive correlation between the reduced AFP susceptibility and increased synthesis of cell wall chitin and β-1,3-glucan in A. niger upon AFP treatment. A slightly different response was observed for P. pastoris. The AFP-resistant wild-type strain responded to the presence of AFP also with a considerably increased level of β-1,3-glucan synthesis (+46%), but, in contrast to the resistant NP1.15 A. niger strain, the chitin level was not changed. However, P. pastoris strain BBA21.3, which expresses the *dtdA* gene and thus is AFP susceptible, responded to AFP treatment with increased (+20%) β-1,3-glucan synthesis, and the chitin level remained constant (Fig. 6C). These data are in agreement with the data observed for the susceptible A. niger wild-type N402 strain. These observations suggest (i) that the A. niger and P. pastoris wild-type strains differ in the strength of their AFP survival responses, (ii) that the defense mechanism against AFP is strong cell wall fortification with both chitin and β-1,3-glucans, and (iii) that this response is somehow hindered by DtdA and/or the unsaturation level of GlyCers.

### The absence of both GlyCer and *dtdA* made A. niger considerably less vulnerable to AFP.

**FIG 7:**
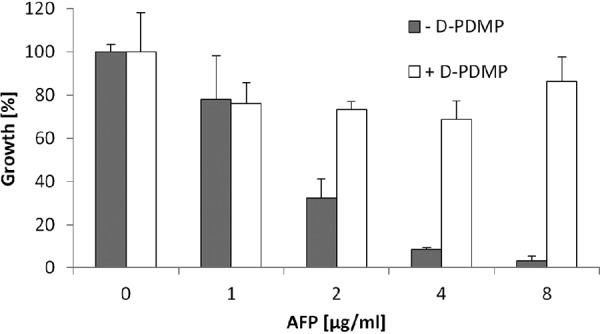
GlyCer-dependent susceptibility of A. niger *dtdA* deletion strain to AFP. Data represent growth of A. niger *ΔdtdA* strain NP1.15 at different AFP concentrations and in the presence (white bars) or absence (gray bars) of the d-PDMP. Growth was evaluated by determining the optical density at 600 nm during microtiter plate cultivations. Data were normalized to the growth, i.e., 100% growth, of control samples which were not treated with AFP. NP1.15 spores were treated with the d-PDMP solvent (water) or d-PDMP. Data are expressed as means of results from two independent biological experiments performed in a technical triplicate. Error bars express standard deviations.

The glucosylceramide synthase inhibitor d-PDMP prevents synthesis of GlcCer in filamentous fungi (23), whereas both GlcCer and GalCer are potential targets of DtdA. We thus analyzed the consequences of depletion of GlcCer mediated by d-PDMP in the *dtdA* deletion strain devoid of unsaturated GlyCer to determine the ability of A. niger to survive AFP. Therefore, *dtdA* deletion strain NP1.15 was treated with d-PDMP, as described above, and the growth rate was assessed in the presence of rising AFP concentrations. As depicted in Fig. 7, the survival of NP1.15 in the presence of d-PDMP improved considerably, by 15% (reaching a final value of 68% growth at 4 μg/ml AFP) to 36% (reaching a final value of 86% growth at 8 μg/ml AFP), compared to the wild-type N402 strain (final values of 53% at 4 μg/ml AFP and 50% at 8 μg/ml AFP; Fig. 2A), suggesting that the unsaturation level of both glycosylceramides, GlcCer and GalCer, defines the susceptibility of A. niger to AFP to a large extent.

## DISCUSSION

This is the first report revealing that fungal GlyCers are key molecules defining the susceptibility of fungi to AFP and that the (un)saturation level of their fatty acid moiety plays a vital role in this relationship. We have interrogated this phenomenon in five different uni- and multicellular fungi and identified a 2-hydroxy fatty N-acyl-Δ3(*E*)-desaturase as the enzyme responsible for the GlyCer desaturation. We named this enzyme DtdA in A. niger. Our genetic, chemical, and bioanalytical data show conclusively that the presence (or absence) of GlcCer makes a fungus AFP sensitive (or AFP insensitive) and that the presence (or absence) of DtdA makes a fungus highly (or minimally) susceptible to AFP. We furthermore provide data which suggest two mechanistic effects that account for this phenomenon and that are not mutually exclusive. Instead, both are in support of the hypothesis of the adopted damage-response framework which we proposed earlier (13) and which is neither AFP centered nor host centered, as follows: (i) unsaturated GlyCers might be (direct or indirect) targets of AFP (see Fig. 2 and 7), and (ii) the presence of saturated GlyCers are a necessary precondition for the proper fortification of fungal cell walls with both chitin and glucans in response to AFP (see Fig. 6). We have shown, using the examples of S. cerevisiae, A. niger, and P. pastoris, that those species differ in the availability of AFP targets (see Tables 1 and 2) and that A. niger and P. pastoris also differ in the strength of their cell wall responses to AFP (see Fig. 6). As nothing is known so far about the overall interplay of (un)saturated GlyCers with AMPs and cell wall synthesizing enzymes, the molecular events have remained elusive and need to be scrutinized in the future.

**FIG 8:**
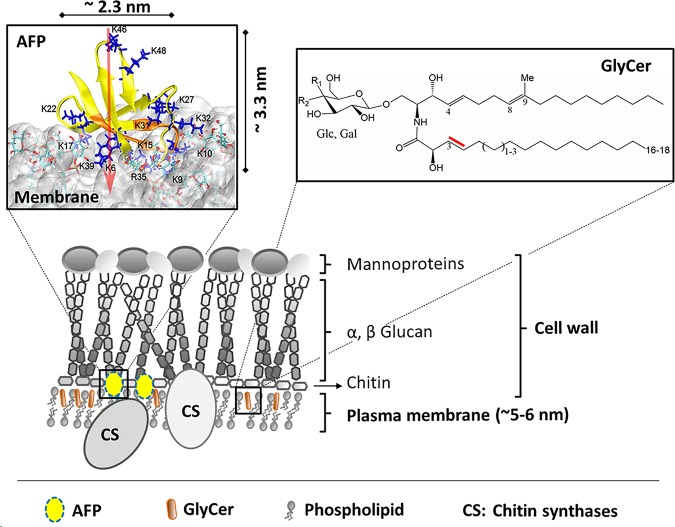
Working model for the mode of action of AFP. See the text for details. Note that the upper left picture is reproduced from Utesch et al. (4), which has been published under Creative Commons Attribution license (CC BY, http://creativecommons.org/licenses/by/4.0/). It displays the MD-simulated interaction of AFP with fungal membrane, including the molecular spatial dimensions of AFP. AFP and its N-terminal γ-core are highlighted in yellow and orange, respectively. Blue sticks, R and K residues; white cloud, fungal model membrane; red arrow, dipole moments of AFP.

GlyCers are synthesized in the endoplasmic reticulum (ER) and Golgi compartment (25, 26) by ER- or Golgi-resident biosynthetic enzymes and are transported in secretory vesicles to the outer leaflet of plasma membranes (25). In mammals, they are known to modulate cell signaling, including calcium signaling and endocytic trafficking (27, 28). In fungi, it has been shown that they differ between various species (29) and that they are important for polarized growth (30), conidiation (21), and stress resistance (31) (for reviews, see references [Bibr B32][Bibr B33][Bibr B34]). Notably, GlyCer levels are quite low compared to those of other membrane lipids and reach only about 0.007% of total lipids in mammalian spleen tissues (35). Although controversially discussed, GlyCers are thought to form, together with proteins, special membrane domains called lipid rafts or ceramide platforms because of their different biophysical properties (36, 37). The colocalization of chitin and glucan synthases within lipid rafts has also been documented for the oomycete Saprolegnia monoica (38). In view of these observations and the AFP knowledge accumulated so far (see the introduction and the present report), we propose the following working model (Fig. 8), which will be studied further in future experiments. We hypothesize that AFP accumulates on the surface of the outer leaflet of fungal plasma membranes, as predicted by recent molecular dynamics modeling approaches (4), which confirmed electron and light microscopy data published earlier (8, 10, 11). AFP interacts via its γ-core motif either directly or indirectly with (un)saturated GlyCers embedded in the outer leaflet. We propose that this interaction is confined to specific membrane regions. This assumption brings together data representing the specific biophysical properties of GlyCers (36, 37) with electron microscopic observations which were made earlier and which clearly demonstrated that AFP accumulates at distinct areas at the plasma membrane and cell wall only in the AFP-sensitive fungus A. niger and not in the AFP-resistant fungus P. chrysogenum (10). These domains might harbor CHSs of classes III and V, which are additional known targets of AFP and are exclusively found in filamentous fungi (8). Assuming that unsaturated GlyCers are important for the proper embedding of class III and class V CHSs in these microdomains, a *dtdA* deletion would expose fewer of these CHSs to AFP, being less susceptible. If challenged with AFP, other chitin and glucan synthases could potentially become activated by the cell wall integrity and calcium signaling pathways, thus fortifying the cell wall to be stronger in a *dtdA* deletion background than in the wild-type background. Note that an interaction of AFP with GlyCers remains to be shown; however, the importance of GlyCers for defining the sensitivity of fungi to plant, insect, and fungal AMPs has already been documented in the literature for a few AMPs (see references 39–42 and Table S1 in the supplemental material). This implies that GlyCers could interact directly with AFP or that GlyCers are important to embed CHSs in the plasma membrane or both. It was shown recently that plant-derived AMP Psd2 interacts preferentially with mimetic membrane domains enriched with GlyCers (43). Previous studies already showed that a change of membrane fluidity can disrupt membrane protein organization by segregating peripheral and integral proteins and thereby interfering with cell wall integrity (44).

10.1128/mSphere.00741-19.6 TABLE S1 AMPs described as associated with GlyCer. Protein models were created with SWISS-MODEL (39), showing the positions of cysteines in yellow. Download Table S1, PDF file, 0.2 MB. Copyright © 2019 Paege et al. 2019 Paege et al. This content is distributed under the terms of the Creative Commons Attribution 4.0 International license.

In conclusion, this report reveals that GlyCers are important determinants of the species specificity of AFP, suggesting that they can be generally viewed as excellent fungus-specific targets for future antifungal drug development programs. We have revealed that a minor structural difference in these molecules—the saturation level of their fatty acid chain, controlled by a 2-hydroxy fatty N-acyl-Δ3(*E*)-desaturase—represents a key to understanding the inhibitory activity of AFP. Future work will disclose how AFP-mediated inhibition of chitin synthesis is linked with the function of GlyCers for the plasma membrane and thus with cell wall integrity.

## MATERIALS AND METHODS

### Strains and cultivation conditions.

Fungal strains used in this study are listed in Table 3. S. cerevisiae and P. pastoris were cultivated at 30°C in yeast extract-peptone-glucose medium (YPG; 0.3% yeast extract, 2% peptone, 1% glucose, 0.5% NaCl), buffered complex glycerol medium (BMGY; 1% yeast extract, 2% peptone, 100 mM potassium phosphate [pH 6.0], 1.34% yeast nitrogen base, 4*10E-5% biotin, 1% glycerol) or methanol medium (BMMY; 1% yeast extract, 2% peptone, 100 mM potassium phosphate [pH 6.0], 1.34% yeast nitrogen base, 4*10E-5% biotin, 0.5% methanol) (45). F. graminearum strains were cultivated at 28°C in minimal medium (MM) or complete medium (CM [MM supplemented with 1% yeast extract and 0.5% Casamino Acids]) (45). A. niger strains were cultivated at 18°C, 30°C, or 37°C in MM or CM (supplemented with 10 mM uridine if required). Transformations of A. niger, selection procedures, genomic DNA extractions, and diagnostic PCRs were performed using protocols described recently (46). A. niger was transformed using a previously described polyethylene glycol (PEG) method (47). Standard PCRs, general cloning procedures, and Southern analyses were carried out according to established protocols (48). Yeast strains were transformed via electroporation, as described previously (49, 50). P. pastoris protein production was performed in adapted MM (1.34% yeast nitrogen base [YNB], 0.5% methanol, 4*10E-5% biotin) at 30°C. Chemically competent Escherichia coli Top10 (Invitrogen) cells were transformed using an established heat shock procedure (48) with the desired plasmid or ligation mix followed by cultivation at 37°C on LB medium containing antibiotic.

**TABLE 3:** Fungal strains used in this study

Species	Strain name(s)	Description	Reference or source
A. niger	N402	*cspA1*	60
A. niger	AB4.1	*cspA1*, *pyrG*	60
A. niger	NP1.15, NP1.16	*cspA1*, Δ*An01g09800*::*AopyrG*, N402 derivative	This work
A. fumigatus	ATCC 9197	Wild type	61
F. graminearum	8/1	Wild type	62
F. graminearum	Δ3FgKO	Δ*FJ176923*	19
P. pastoris	GS115	*his4*	63
P. pastoris	BBA21.3	GS115 transformed with pNP1.13	This work
P. pastoris	NP7.1	GS115 transformed with pPIC3.5	This work
P. pastoris	SZ51	pGAPZ/C + *FJ176923*	This work
S. cerevisiae	BY4741	*MAT***a** *his3*Δ*1 leu2*Δ*0 met15*Δ*0 ura3*Δ*0*	64
S. cerevisiae	BBA20.2	BY4741 transformed with pBBA 5.1	This work

### Molecular cloning.

In order to clone the cDNA of the predicted Δ3(*E*)-desaturase from A. niger (An01g09800), total RNA from a culture of A. niger strain N402 was extracted from its biomass following the TRIzol method (51) and treated with DNase using an Ambion DNA-free kit (Invitrogen). cDNA was generated from total RNA using a Thermo Fisher RevertAid H Minus first-strand cDNA synthesis kit with an oligo(dT)_18_ primer, according to the manufacturer’s instructions. An01g09800 cDNA was used as the PCR template for the Q5 proofreading polymerase (NEB). Sequence alignments of the PCR product confirmed the predicted intron-exon boundaries of An01g09800. This PCR product also served as a template for a PCR to amplify an open reading frame fragment for sticky-end ligation into plasmid pPIC3.5 (52) restricted with EcoRI and NotI. The resulting plasmid, pNP1.13, was linearized with SacI and transformed into P. pastoris strain GS115 (Invitrogen) via electroporation, giving strain BBA21.3. Total cDNA from F. graminearum strain 8/1 (19) was used as a template for the amplification of the Δ3(*E*)-desaturase FJ176923 gene with *Pwo* polymerase (Peqlab) and was ligated into vector pGEM-T (Promega). After restriction performed with EcoRI and NotI, the corresponding fragment containing Δ3(*E*)-desaturase was ligated into pGAPZ/C (Invitrogen), leading to production of the plasmid, which, after linearization with BglII, was transformed into P. pastoris strain GS115, resulting in strain SZ51. The cDNA of the Δ3(*E*)-desaturase An01g09800 gene was further used as a template to amplify a fragment for Gibson assembly with fragments of the S. cerevisiae TEF promoter and CYC terminator into vector pYIP5 (Addgene) restricted with HindIII and SalI. PCR amplification of the cassette via Q5 polymerase for sticky-end cloning into vector pRS306 (Addgene) after restriction performed with the XhoI and XbaI enzymes led to formation of plasmid pBBA5.1, which was transformed into S. cerevisiae strain BY4741 (53) via electroporation. The resulting strain was named BBA20.2. All DNA constructs were verified by sequencing before transformation. Quantitative PCR (qPCR) was carried out using Maxima sybr green/ROX qPCR master mix (Thermo Fisher), following the manufacturer’s instructions. Up to 500 ng cDNA was used as a template. The actin gene served as a control (54).

### Identification and deletion of the A. niger Δ3(*E*)-desaturase gene.

The Δ3(*E*)-desaturase sequence of F. graminearum FJ176922.1 (19) was used as a reference to identify the orthologue in A. niger via pBLAST (55). The identified open reading frame, An01g09800, displayed sequence identity of 61% on the amino acid level to FJ176922.1. We used the split marker approach (56) with about 500 ng gel-purified PCR product each to replace An01g09800 with p*yrG* via a crossing-over event. The forward bipartite was created using primers 677 and 462 (Table S2) with the upstream regions of *An01g09800* and *pyrG* as the templates. The reverse bipartite was amplified with primers 461 and 680 (Table S2) using *pyrG* and the *An01g09800* downstream fragment as the templates. A. niger AB4.1 was transformed with both bipartites, and positive transformants were selected due to uridine prototrophy (*pyrG*). After two purifications performed on MM, genomic DNA of the transformants was tested via PCR using primers 677 and 716 (Table S2). Southern blot analysis using SalI for the fragmentation of genomic DNA and a digoxigenin-labeled DNA probe binding fragments containing *pyrG* confirmed deletion of *An01g09800* in two strains, which we named NP1.15 and NP1.16, respectively.

10.1128/mSphere.00741-19.7 TABLE S2 Primers used in this study. Sequences homologous to the A. niger genome are given in uppercase letters, and letters in lowercase refer to sequences introduced for PCR/cloning purposes. Download Table S2, PDF file, 0.3 MB. Copyright © 2019 Paege et al. 2019 Paege et al. This content is distributed under the terms of the Creative Commons Attribution 4.0 International license.

### Purification and analysis of GlyCer.

The biomasses of the A. niger and P. pastoris strains were frozen in liquid nitrogen, freeze-dried, and weighed. A total of 0.7 g dry biomass was ground and extracted twice with 20 ml chloroform and methanol (2:1 [vol/vol]), subjected to vortex mixing with glass beads, and gently shaken for 2 h. A total of 1 ml demineralized water was added before centrifugation was performed (340 × *g*, 4 min). The supernatant was mixed with 7 ml of 0.75% NaCl solution mixed in water and centrifuged before the bottom phase was separated, and the organic phase (covered by argon as an inert gas) was evaporated at 50°C. The sample was resuspended in 1 to 3 ml chloroform and methanol (2:1 [vol/vol]) and filtered through a cotton-plugged Pasteur pipette. Samples (5 μl each) were analyzed via thin-layer chromatography (TLC) using silica gel plates and a runtime of 1 h in chloroform and methanol (85:15 [vol/vol]). The GlcCer from P. pastoris was applied as a standard. Visualization of the samples under UV light via the use of 8-anilinonaphthalene-1-sulfonic acid followed. The final detection was carried out with alpha-naphthol sulfuric acid and heating to 170°C. The GlyCer-positive samples were used for subsequent preparative TLC to purify the GlyCer from the lipids extracted. Bands containing the GlyCer were scratched from the TLC plates and suspended in 6 ml chloroform and methanol (2:1 [vol/vol]), and 1.5 ml of 0.75% NaCl mixed in water was then added. After the centrifugation and separation of the organic phases, the samples were filtered through cotton-plugged Pasteur pipettes until all remaining solids had been removed and the liquid was evaporated at 50°C using argon gas. Samples were reconstituted in 100 μl chloroform and methanol (2:1 [vol/vol]) and stored at –20°C until they were analyzed.

### Mass spectrometry of GlyCer.

A total of 1 μl of purified GlyCer solution or peptide calibration standard (8206195; Bruker Daltonics) was sandwiched in 0.5 μl α-cyano-II-hydroxycinnamic acid on a matrix-assisted laser desorption ionization (MALDI) ground steel target (Bruker). Acquisition of MALDI spectra was performed using an ultraflex III mass spectrometer (Bruker Daltonics) in reflector mode, detecting positively charged ions in the range of 400 to 4,000 *m/z*. Each sample was analyzed for the expected signals at 778 *m/z* and 776 *m/z* for saturated GlyCer and unsaturated GlyCer, respectively. In addition, the instrument was operated in MS/MS mode for the parental ion masses of 777 and 778 as mentioned above. The evaluation of the spectra was carried out using the program “mMass—Open Source Mass Spectrometry Tool” (57).

### Chitin and β-1,3-glucan quantification.

P. pastoris strains were inoculated from preculture grown in BMMY medium for 24 h and incubated in fresh BMMY medium for 48 h. The methanol was supplemented every 24 h to induce the expression of the Δ3(*E*)-desaturase. A total of 50 μg/ml AFP was added at the start of the cultivation when needed. A total of 10E6 spores/ml (which is 3 orders of magnitude higher than the level that was used for the susceptibility assay) from the A. niger N402 (wild-type) strain were cultivated in 100 ml YPG medium at 28°C for 24 h. A total of 20 μg/ml AFP was added, and the cultivation was maintained for up to 72 h. Biomass was harvested via centrifugation (3,090 × *g*, 10 min) or by filtration (Sartorius; format 3 hw), freeze-dried, and used directly for the analysis of β-1,3-glucan content according to a previously described method (58), with the exception that an ultrasonic bath was used for 1 h instead of ultrasonic treatment for 30 s. The culture supernatant was discarded. The chitin content was measured according to a previously described method (59). In brief, 50 mg freeze-dried biomass was used for the cell wall extraction. A total of 4 mg of freeze-dried cell wall fraction was used for the chitin extraction. Calibration curves were generated with curdlan for β-1,3-glucan and with glucosamine for chitin. Purified β-1,3-glucan or chitin was quantified using fluorescence spectroscopy (excitation wavelength, 392 nm; emission, 502 nm) or photometry at 520 nm.

### Susceptibility assays.

The AFP susceptibility assay was performed to determine the MIC as described previously (8, 13). Susceptibility to AFP in the presence or absence of 35 μM d-threo-1-phenyl-2-decanoylamino-3-morpholino-1-propanol (d-PDMP) dissolved in Milli-Q water was determined in the same way. All experiments were performed twice with technical triplicates.
