# Supplementary figures and images for: Species-Specific Differences in the Susceptibility of Fungi to the Antifungal Protein AFP Depend on C-3 Saturation of Glycosylceramides
Source: mSphere. 2019 Dec 11;4(6):e00741-19. doi: 10.1128/mSphere.00741-19 (PMC6908424; doi:10.1128/mSphere.00741-19)

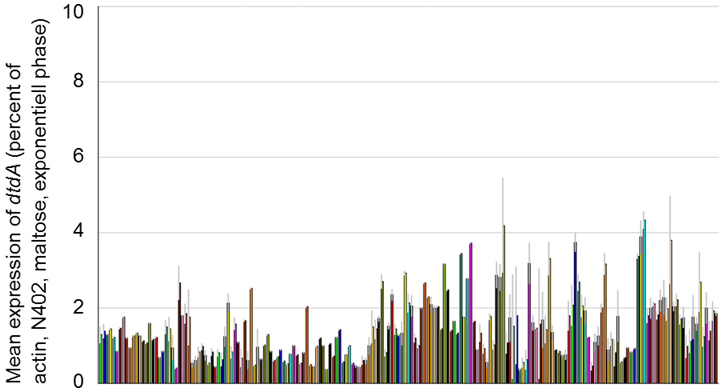

Supplement: FIG S1 [file mSphere.00741-19-sf001.tif]

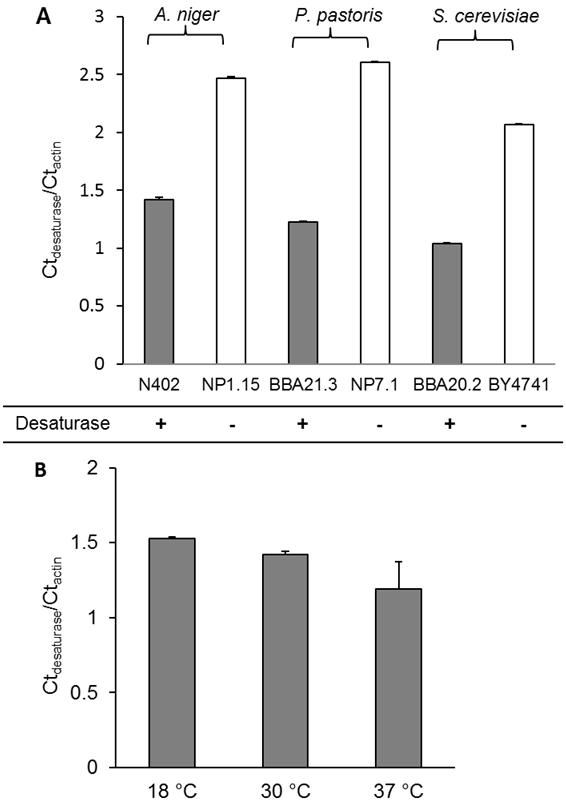

Supplement: FIG S2 [file mSphere.00741-19-sf002.tif]

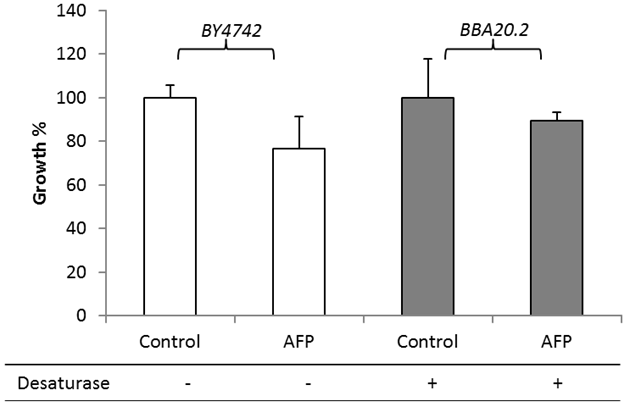

Supplement: FIG S3 [file mSphere.00741-19-sf003.tif]

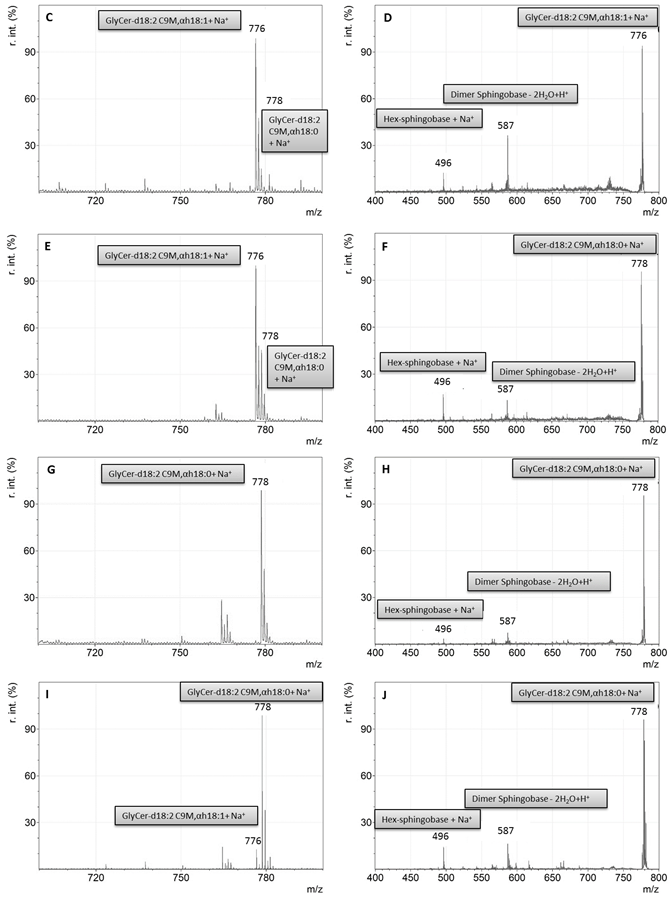

Supplement: FIG S4 [file mSphere.00741-19-sf004.tif]

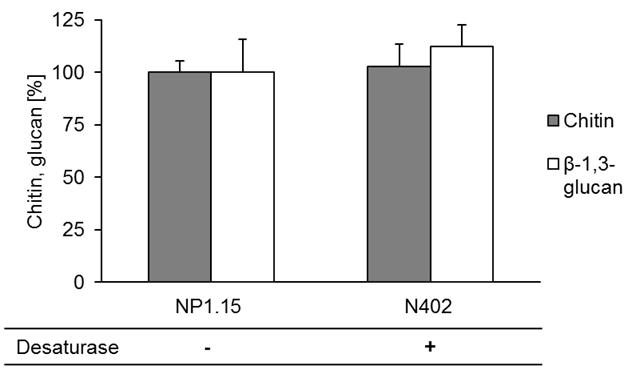

Supplement: FIG S5 [file mSphere.00741-19-sf005.tif]
