# Supplementary material for: Species-Specific Differences in the Susceptibility of Fungi to the Antifungal Protein AFP Depend on C-3 Saturation of Glycosylceramides
Source: mSphere. 2019 Dec 11;4(6):e00741-19. doi: 10.1128/mSphere.00741-19 (PMC6908424; doi:10.1128/mSphere.00741-19)
Supplement: TABLE S1 [file mSphere.00741-19-st001.pdf]

Table S1

Name	Organism	Structure	Uniprot-no.	Source
RsAFP	*Raphanus sativus*	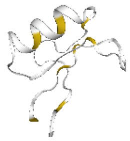	P69241	(41)
Psd1	*Pisum sativum*	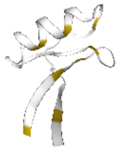	P81929	(20)
Psd2	*Pisum sativum*	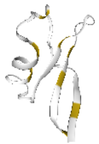	P81930	(43)
DmAMP1	*Dahlia merckii*	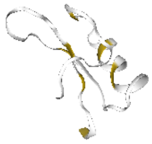	P0C8Y4	(40)
Heliomicin	*Heliothis virescens*	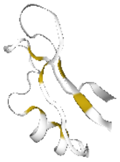	P81544	(41)
*Ms*Def1	*Medicago sativa*	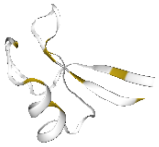	Q9FPM3	(42)
PAF	*P. chrysogenum*	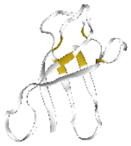	D0EXD3	(30)
AFP	*A. giganteus*	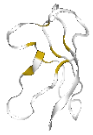	P17737	this work
