# Supplementary material for: Species-Specific Differences in the Susceptibility of Fungi to the Antifungal Protein AFP Depend on C-3 Saturation of Glycosylceramides
Source: mSphere. 2019 Dec 11;4(6):e00741-19. doi: 10.1128/mSphere.00741-19 (PMC6908424; doi:10.1128/mSphere.00741-19)
Supplement: TABLE S2 [file mSphere.00741-19-st002.pdf]

Table S2

Primer Code	Localization	Orientation	Sequence
459	5`end *pyrG* region	fw	aagccgctgctggaattgTGTAAAACGACGGCCAGT
460	3`end *pyrG* region	rev	CGATGGATAATTGTGCCGTGT
461	5`end *pyrG*	fw	ATTGACCTACAGCGCACGC
462	In *pyrG*	rev	CCGGTAGCCAAAGATCCCTT
677	Upstream *An01g09800*	fw	ACACATGGGAGGGGTAATGA
678	Upstream *An01g09800* with *AopyrG* start	rev	tcactggccgtcgttttacaCATGTTATCCGACCCATCCC
679	Downstream *An01g09800* with *AopyrG* end	fw	acacggcacaattatccatcgTCTCACTAGCAGACGAGGTT
680	Downstream *An01g09800*	rev	ACCCATCGATTAGGGAGAGG
716	Downstream	rev	TCACCACCACAATCTACCCC

	680		
936	pYip5 with 5` site *tef*	fw	gctgatgagctttaccgcagCAAAATGTTTCTACTCCTTT
937	3`site *tef* with 5`site Kozak sequence Yeast and *An01g09800*		gggtccatggtggcggcgggCTTAGATTAGATTGCTATGC
938	*An01g09800* with 3`end *tef* Kozak sequence yeast	fw	aatctaagcccgccgccaccATGGACCCTTCCACCTTTATexon
939	3`site *An01g09800* with 5`end *cyc*	rev	tgacataactaattacatgaCTAAACTTCCGCTTGATTTC
940	5`end *cyc* with 3`end *An01g09800*	fw	gaaatcaagcggaagtttagTCATGTAATTAGTTATGTCA
941	3` end *cyc* with pYIp5	rev	caccgaaacgcgcgaggcagAGCGTCCCAAAACCTTCTCA
1436	5`end	fw	gatccacataATGGACCCTTCCACCTTTAT

	An01g09800 with BamHI site		
1439	*5`An01g09800*	fw	ATGGACCCTTCCACCTTTAT
1440	*3`An01g09800*	rev	TCAGCTAAACTTCCGCTTGA
1443	*3`An01g09800 with* EcoRI site	rev	aattcTCAGCTAAACTTCCGCTTG
1529	*3`An01g09800 with* NotI site	rev	gcgaattaattcgcggccgcTCAGCTAAACTTCCGCTTGA
1530	*5`An01g09800 with* EcoRI	fw	gatcctacgtagaattcattATGGACCCTTCCACCTTTAT
